# National Climate Change Risk Assessments to inform adaptation policy priorities and environmental sustainability outcomes: a knowledge systems perspective

**DOI:** 10.1007/s10584-022-03464-2

**Published:** 2022-12-21

**Authors:** Iain Brown, Pam Berry

**Affiliations:** 1 Dept. of Geography and Environmental Science, University of Dundee, Dundee, DD1 4HN UK; 2 Environmental Change Institute, School of Geography and the Environment, University of Oxford, Oxford, OX1 3QY UK

**Keywords:** Climate change, National Climate Change Risk Assessments, National Adaptation Plans, Environmental sustainability, Knowledge systems

## Abstract

National Climate Change Risk Assessments (CCRAs) have a key role in informing priorities for adaptation policy but face significant challenges due to multiple facets of risk and adaptation. Issues are especially pronounced for meeting goals of environmental sustainability due to the complex dynamics of socio-ecological systems. In practice, a CCRA can therefore differ from its original conceptual blueprint. These challenges are explored from a knowledge systems perspective, focusing on the role of stakeholders/policymakers, risk descriptors, methods, evidence sources, and scientists. A UK case study evaluates recent developments (CCRA3) including identification of policy urgency through adaptation shortfalls and its application to the natural environment. Important science-policy issues are also highlighted regarding inclusion of opportunities, systemic risks, residual risks, and risk tolerance. A general conclusion is that CCRAs inevitably leave open questions which lead back to their evolving role in the science-policy interface. A knowledge systems perspective identifies CCRAs as open, adaptive, reflexive processes that help redefine interpretations of risk and adaptation, rather than just providing a specific policy-relevant product. This perspective identifies scope for progressive refinement of CCRAs to enhance collective science-policy adaptive capacity whilst also engaging wider society. For environmental sustainability, this open process can be used to iteratively redefine robust future pathways and system reference conditions that also better reflect evolving societal perceptions and tolerance on sustainability risk in the face of climate change.

## Introduction

Climate change and environmental degradation are increasingly recognised as topmost global priorities, fundamentally linked for human wellbeing in terms of sustainability risks (Pörtner et al. 2021; Wassénius and Crona 2022). Environmental sustainability objectives thus prioritise development pathways that protect the long-term integrity and functioning of biodiversity and ecosystems on which humans depend (Kuhlman and Farrington 2010). However, no country is currently on track to deliver basic human needs within a globally sustainable level of natural resource use (Fanning et al. 2021). Expeditious actions to resolve this dichotomy are challenged by the complexity of socioecological systems, differential values, and knowledge-related impediments that hinder forward planning and proactive decision making. Challenges are compounded by additional unmitigated risks from anthropogenic climate change because identifying and implementing effective adaptation responses is complicated by multi-faceted, cross-scalar relationships between risk and adaptation (Craft and Fisher 2018; New et al. 2022).

At country-level, adaptation policy development is commonly scoped through climate change risk assessments (CCRAs) to inform National Adaptation Plans (NAPs) using identification and prioritisation of the most significant climate-related risks (European Environment Agency 2018; USGCRP 2018; Ministry for the Environment 2019; Feng and Chao 2020; Song and Lee 2021). NAPs define strategies to meet identified adaptation needs and are now included in a global adaptation goal (Morgan et al. 2019). However, utility of CCRAs, and their role in stimulating policy, has been debated, especially when derived from conventional risk assessments not intended to handle inherent uncertainties and complexities that characterise climate-related risks (Adger et al. 2018; Smith et al. 2022). Current NAPs have also been found to have important limitations, most being high-level aspirational documents without detailed actions required to deliver effective responses (Lesnikowski et al. 2016; Lee et al. 2022).

Current difficulties in matching recognised climate risks with effective adaptation responses highlight the need for improved two-way communication between national CCRAs and NAPs, although CCRAs can be developed at any governance scale. In recent years, climate change science has moved towards common understanding of risk as a source of potential harm or disruption to something of value in human or ecological systems, complemented by conceptual framing based on combined factors of hazard, exposure, and vulnerability (Reisinger et al. 2021). This understanding also explicitly aims to recognise the diversity of values and objectives associated with risk and risk perception (Reisinger et al. 2021). General guidance for CCRAs has also evolved, including a recent international protocol (ISO14091: Smith et al. 2022). However, challenges remain in developing a consistent and comprehensive national CCRA across a wide range of disparate risks, including at multiple spatial and temporal scales, and particularly for complex adaptive socioecological systems (Garschagen et al. 2021). Policy-based requirements also increasingly require evidence contextualised against efficacy of adaptation responses to identify priorities for further action informed by magnitude and urgency of the risk (Adger et al. 2018).

To further investigate these issues, the present study employed a knowledge systems perspective to define a generic CCRA template. Knowledge systems aim to elucidate structure and processes connecting knowledge production with user notions of utility and value, as advocated for the sustainability agenda through co-production (Cornell et al. 2013; Fazey et al. 2020; Oliver et al. 2021). This framing acknowledges wider governance contexts, including that policymaking can be subject to many other influences that can disrupt aspirations for coherence and rationality (Rose 2014). Climate change and sustainability are especially difficult policy issues because of long timescales and inherent uncertainties, requiring different strategies than those based on predictability and optimality, or incremental ‘muddling through’ (Hallegatte 2009). Hence, increased demand for scientific advice that is structured and directly relevant to evolving policy agendas. Meeting this demand is usually a messy, iterative progression towards establishing a common purpose between science and policy, but these exchanges are usually obscured from external view beneath a *horizon of visibility* that only shows final codified knowledge products using clear-cut arguments and legitimised methods (Leith et al. 2018). We therefore aim to explore below this horizon, reconsidering key issues of climate risk and adaptation that national CCRAs need to tackle more explicitly to stimulate further progress. These issues highlight added value of national CCRAs as shared knowledge and learning platforms between science, policy, and practice, rather than isolated products produced every few years.

A UK CCRA case study is used to evaluate recent developments, using the authors’ own involvement as natural environment leads (Berry and Brown 2021), to help explore science-policy issues under the visibility horizon including progress against environmental sustainability goals. This has allowed practical realities of knowledge exchange to be compared against a conceptual blueprint, including generic methodology issues. In this regard, the mode of investigation is analogous with IPCC report authors who have similarly probed lessons learned from the assessment process to-date and hence the way forward (e.g. Mach and Field 2017).

## A CCRA knowledge systems template

**Fig. 1:**
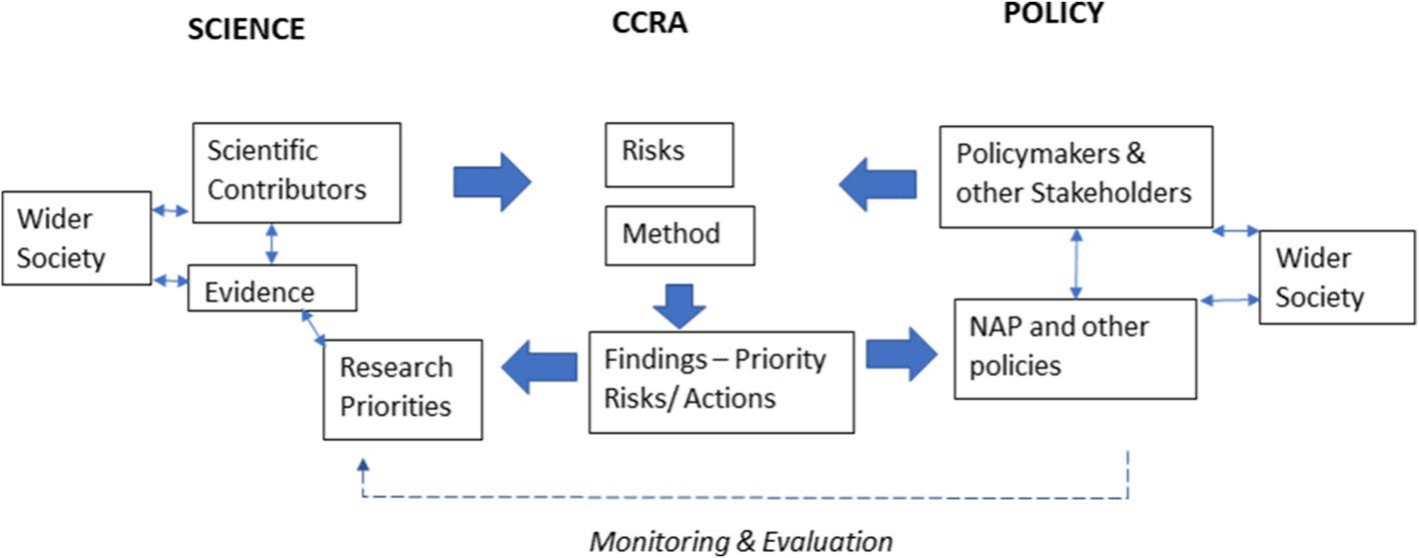
CCRA knowledge system components across the science-policy interface

CCRA knowledge systems should define the role of policymakers and other stakeholders, risk descriptors, methods, evidence, and scientific contributors (Fig. 1). If these features are not systematically integrated, they can pull in different directions, disrupting knowledge flow for adaptation planning. A national CCRA therefore needs to develop a structure and process to facilitate integration, including a methodology to maximise finite resources and target key science-policy leverage points (Adger et al. 2018).

### Policymakers and other stakeholders

Policymakers and other decision makers can be defined as key CCRA stakeholders, based on their assumed requirements for updated assessment of climate risks. For NAPs, predominant policy barriers are limited issue awareness, miscommunication, fragmented decision making, and resource constraints (Lee et al. 2022). Policy-based knowledge needs are usually defined in terms of clear concise messaging synthesising both qualitative and quantitative evidence, but risk assessment and communication also need to avoid problems of oversimplification and reductionism, notably when evidence from complex systems is over-generalised, uncertainty ignored, or contextual framing of risks and decisions omitted (Stirling 2010; Rigg and Reyes Mason 2018).

### Risk descriptors

Risks are key foci for a CCRA, but risk itself has multiple meanings. Hence, characterising risk for policy purposes can be complicated by varying interpretations, both subjective and objective (Krebs 2011; Wassénius and Crona 2022; Smith et al. 2022). Although climate science has evolved a common understanding of risk, differences remain amongst policymakers and wider society that influence risk communication (Tangney 2019). A distinctive feature of these differences is framing and compartmentalisation of individual risks for focal analysis and action. Ideally, a CCRA should establish a consistent and logical framework of risk descriptors. However, theoretical characterisation of risks may differ from how they are interpreted and described in practice, as for example through individual policies or regulations (Adger et al. 2018). Legacy regulations based on specific narrowly-defined risks may be challenged by climate-related modification of the wider system, leading to potential ‘lock-in’ maladaptation effects if unresolved (Brown and Everard 2015). Complications regarding consistency and terminology can also occur by inclusion of opportunities as potential ‘positive risk’ outcomes, as described below for UK CCRA3.

### Method

Various climate risk methods have been developed in different contexts (Jurgilevich et al. 2017; Smith et al. 2022). However, some have been criticised as over-reductive compared to the scale and diversity of climate change (Howarth et al. 2018; Rigg and Reyes Mason 2018). Hence, a generic national CCRA methodology needs to be able to cover a wide range of climate-related risks without biases, reflecting general issues of consistency and prioritisation in policymaking. An increasingly important role for CCRA methods is evaluating adaptation progress, also including issues of consistency, comparability, comprehensiveness, coherency, and clarity on adaptation objectives (Berrang-Ford et al. 2019; Singh et al. 2021).

Complexity and inherent uncertainty mean conventional risk methods based on objective predictability of probability and consequences are unsuited to CCRAs, whilst models and empirical data need to be interpreted contingent on underlying assumptions (Adger et al. 2018). For example, climate models are key tools to interpret changing risk, but necessarily define some parameters based upon expert opinion rather than empirical data alone, nor can model design cover all earth system uncertainties (Weaver et al. 2013).

Furthermore, conceptualisation of risk in climate science as applicable to ‘something of value’ (Reisinger et al. 2021), recognises varying subjective interpretations. Methodological choices therefore also have an ethical dimension, requiring transparency on value judgements, especially regarding issues associated with the natural environment, sustainability goals, and societal inequalities (Brown 2018). Some national CCRAs therefore have put particular emphasis on a pluralistic values-based approach (e.g. Aotearoa New Zealand: Ministry for the Environment 2019). Taken together, all of these contextual issues generally point towards methods applied through a narrative-based approach, complemented by quantified statements wherever applicable and available.

### Evidence

National CCRAs need to sift an often-extensive evidence base for policy relevance, whilst interpreting it consistent with method and risk descriptors. A key requirement is for causal inferences on attribution of risk factors referenced against adaptation objectives and hence, where possible, their effectiveness regarding risk management. These requirements generally necessitate a mix of theoretical, modelling, and empirical evidence sources, as synthesised and assessed through systematic review (Song and Lee 2021), with consensus derived from multiple independent evidence sources leading to higher assessment confidence (Mach et al. 2017). However, consistency challenges have also suggested increased use of formalised review procedures (Berrang-Ford et al. 2015).

### Scientific contributors

As with IPCC reports, national CCRAs are delivered by teams of scientific contributors through integration of specialist knowledge. Use of expert judgement to assess evidence sources and derive summary conclusions on significant cause-effect relations is an established mechanism, although procedures vary (Mach et al. 2017; Borie et al. 2021). In particular, CCRA contributors are required to interpret multiple forms of evidence consistent with risk descriptors, methodology, and policy contexts, including capability to recognise emergent risks at the limit of current knowledge. Limited or contradictory evidence can raise contentious issues, with draft reports potentially undergoing multiple iterations based on reviewer or stakeholder feedback and dialogue, impinging on time availability. A large team of contributors can potentially provide more expertise but increase logistical overheads.

## Case study: the UK CCRA

The UK is distinctive due to statutory government requirements (Climate Change Act 2008) to produce a CCRA every five years to inform development of its NAP. Each CCRA cycle comprises independent scientific assessment of the evidence, then a government response leading to a new NAP (Porter and Clark 2023). This process is now in its third cycle following publication of the CCRA3 evidence report and government response (Betts et al. 2021; HM Government 2022). Following the second cycle, despite the UK government accepting nearly all CCRA2 recommendations, the resulting NAP was criticised for being too general, lacking detailed actions (Climate Change Committee 2021).

The UK CCRA aims to integrate scientific knowledge, formal review, and stakeholder representation, albeit more intensively compared to some other countries (cf. Song and Lee 2021). Primary focus for knowledge exchange has been on government and its agencies, but with some wider stakeholder engagement (Watkiss and Betts 2021). Each CCRA cycle has aimed to learn and improve on previous versions, within resource constraints. CCRA1 conducted a preliminary assessment of a large number of individual risks, with a subset of higher magnitude risks assessed in more detail. However, quantification of climate risks using response metrics to relate risk magnitudes to specific climate parameters was generally found too reductive (Warren et al. 2018), particularly for the natural environment due to the complexity of cause-effect relations, including non-climate factors (Semenov et al. 2012; Brown 2015). CCRA2 methodology therefore aimed for a more overt policy focus based on urgency of actions (Warren et al. 2018) which is also mainly consistent with ISO14091 guidance (Smith et al. 2022). This was subsequently refined for CCRA3 to include the effectiveness of existing adaptation responses (Watkiss and Betts 2021; Sect. 4.3). As CCRA2 was a smaller exercise, it was largely based upon the CCRA1 priority risk subset, although some risks were redefined or merged, and for the natural environment a common framework linking biodiversity with ecosystem services was utilised to provide consistency (Brown 2018). For CCRA3, risk descriptors were further refined as a joint science-policy exercise (Section 4.2).

The present article particularly draws upon findings from the CCRA3 natural environment chapter (Berry and Brown 2021), for which the number of risks assessed as requiring urgent policy action increased compared to CCRA2. This was a consequence of both improved evidence on changing risk magnitudes and limited evidence for adaptation responses in moderating risks (Table 1). Assessment of opportunities identified them primarily as knowledge gaps constraining supportive actions, and hence as research priorities. CCRA3 overall implies a major shortfall regarding environmental sustainability, despite this not being a pre-defined assessment objective.

**Table 1:** UK CCRA3 summary for the natural environment (Berry and Brown 2021) (*L*, low; *M*, medium; *H*, high)

*Risks (R) and opportunities (O)*	Present inherent risk (confidence)	Future inherent risk* (confidence)	Adaptation shortfall (confidence)	Urgency assessment (confidence)
*Terrestrial species and habitats (R)*	H (H)	H H H H (M)	YES (L/M)	Further action (M)
*Terrestrial ecosystems PPI*^*1*^* (R)*	M (M)	H H H H^*2*^ (M)	YES (M)	Further action (M)
*Terrestrial species and habitats (O)*	M (M)^*3*^	M M M H (M)	YES (L)	Research priority (L)
*Soils (R)*	M (M)	H H H H (L)	YES (L)	Further action (L)
*Carbon stores and GHG emissions (R, O)*	M (M)^*4*^	H H H H (L)	YES (L)	Further action (L)
*Agriculture and forest productivity (R)*	M (H)	H H H H (M)	YES (L)	Further action (M)
*Agriculture PPI*^*1*^* (R)*	M (H)	H H H H (L)	YES (M)	Further action (M)
*Forestry PPI*^*1*^* (R)*	M (H)	M/H H H H (L)	YES (L)	Further action (M)
*Agriculture and forest productivity (O)*	M (L)	M/H H H H (L)	YES (L)	Research priority (L)
*Saline intrusion to water (R)*	L (H)	L L L ? ^*5*^ (L/M)	YES (M)	Research priority^*6*^ (L/M)
*Freshwater species and habitats (R)*	M (M)	M M M M^*7*^ (M)	YES (L)	Further action (M)
*Freshwater ecosystems PPI*^*1*^* (R)*	M^*8*^ (H)	M M M H^*9*^ (M)	YES (L)	Further action (M)
*Freshwater species and habitats (O)*	L (L)	L L L L (L)	YES (L)	Sustain current action (L)
*Marine species and habitats (R)*	M (M)	H H H H (L)	YES (L)	Further action (M)
*Marine ecosystems PPI*^*1*^* (R)*	M (M)	H H H H (L)	YES (L)	Further action (L)
*Marine species and habitats (O)*	M (L)	M/H H H H (L)	YES (L)	Research priority (L)
*Coastal species and habitats (R, O)*	M (M)^*10*^	H H H H (L/M)^*10*^	YES (L)	Further action (M or L/M ^*11*^ or L^*12*^)
*Landscape character (R, O)*	M (L)^*13*^	M/H H H H (L)	YES (L)	Research priority (L/M)

^*^Defined for 2050 on a 2 °C and 4 °C pathway and 2100 on a 2 °C and 4 °C pathway respectively

^1^*PPI*, pests, pathogens, and invasive non-native species; ^2^England only (otherwise M M M H); ^3^(H) for Scotland; ^4^(L) for Scotland; ^5^L L L L for Scotland and Northern Ireland; ^6^England and Wales only (otherwise Watching Brief); ^7^ M M M M for Northern Ireland; ^8^H for England; ^9^H H H H for England; ^10^(L) for Northern Ireland; ^11^Scotland; ^12^Northern Ireland; ^13^ (M) for Wales

## UK CCRA3 knowledge system and the natural environment

### Policymakers and other stakeholders

CCRA3 was developed during a period of major UK policy transition following exit from the European Union and further influenced by the COVID-19 pandemic. This transition has included increased recognition of environmental sustainability goals for long-term policy development and the need to reframe economic criteria consistent with such goals (Defra 2018; Dasgupta 2021).

Stakeholder engagement occurred at various stages, including for risk descriptors (Sect. 4.2), methodology (Sect. 4.3), evidence (notably non-academic reports), and feedback on working drafts. Face-to-face meetings during stakeholder workshops were a key mechanism for focussed discussion on key issues, including relationships between method, evidence, and risks, but were disrupted by the COVID-19 pandemic. Workshops also discussed current policy progress and additional adaptation options. Review comments on report drafts were logged and subsequently published to show transparency of process (UK Climate Risk 2021), including issues of controversy or dispute regarding both science and policy interpretation.

For the natural environment, consistent with other recent studies (e.g. Lee et al. 2022), adaptation policy actions were generally found vaguely defined, strategies remaining primarily aspirational with details limited on actual responses. Policies included in the second UK NAP were often existing sectoral policies collated under an adaptation theme, including frequent reference to needs for increased ‘climate resilience’ but rather limited use of resilience concepts to co-ordinate responses consistent with a sustainable transformation (e.g. diversity, redundancy, modularity, self-organisation: cf. Walker 2020). Instead, resilience was apparently associated with objectives (often undeclared) to maintain systems properties in their current form incurring an increased likelihood that this would perpetuate underlying systems vulnerabilities. For example, on the coast, despite ongoing sea-level rise and new evidence indicating future rises may be greater than previously assessed, the current position remains dominated by hard adaptation responses (coast defence structures) and local ‘hold the line’ policies that in many locations acts to lock-in future vulnerability and further degrade coastal ecosystems (Brown 2022).

Another general CCRA3 finding was that monitoring, evaluation, and progress reporting of UK adaptation policies remain very limited, even when policy aspirations were clearly associated with target outcomes. This is despite such procedures being good policymaking practice (HM Treasury 2020) and foundational for implementing resilience-based strategies (Walker 2020). Limited adaptation monitoring highlights conceptual, analytical, and practical barriers in the science-policy interface, because potentially systematic monitoring to assess both changing risks and effectiveness of adaptation actions could be provided by the scientific community (e.g. Pearce-Higgins et al. 2022).

### Risk descriptors

UK CCRAs have been structured around specific risks, meaning risk descriptors represent an important influence on assessment. Successive CCRAs have defined risks differently, complicating comparative assessment, but reflecting an evolving science-policy dialogue. CCRA3 returned to policymakers as its key audience to elicit a list of policy-relevant risk descriptors, further refined through structured dialogue into a final agreed list of risks and opportunities.

The CCRA3 approach aimed to recognise that policy engagement in defining risks could facilitate greater sense of ‘ownership’ in the assessment results, including improved referencing to decision contexts. However, this did incur some anomalies requiring further dialogue, perhaps most notably when the assessment team considered risks were too narrowly-defined scientifically. Hence, for the natural environment, ‘risks from invasive non-native species’ and ‘risks from existing pests and pathogens’ were originally separate risks, primarily due to current legislation definitions, but evidence suggested climate change blurs distinctions, and it would be most meaningful to group them together. Similarly, original policy focus on risks to natural carbon stores was expanded to include all biogenic greenhouse gases in terms of inter-related risks and opportunities (e.g. land use decisions). Coastal erosion and flooding were also originally separate policy risks but subsequently grouped together as inter-related cause-effect processes (cf. Pollard et al. 2019). In each of these cases, systems-based evidence interpretation suggested merged risk descriptors would be most consistent with coherent adaptation policy objectives.

Such developments are indicative of natural environment assessment challenges (compared to other CCRA sectors). The complexity of natural systems usually means multiple interacting relationships between climate drivers and potential effects, hence advantages of broad risk groupings to investigate systemic cause-effect relations and associated requirements for integrated actions to address consequences. CCRA3 similarly found that distinctions between risks and opportunities can be context or pathway dependent (Sect. 5.1). Hence, to reconcile scientific validity with policy relevance, it may be more suitable to define broad risk descriptors together with hierarchical sub-categories matching specific policy definitions.

More discussion of risk descriptor hierarchies could therefore provide a good strategic focus for further science-policy engagement, particularly when mapped onto current policy development for environmental sustainability. For example, CCRA3 dialogue indicated that further developments could usefully explore an integrated risk management structure using the ecosystem services framework (Section 5.2).

### Method

**Fig. 2:**
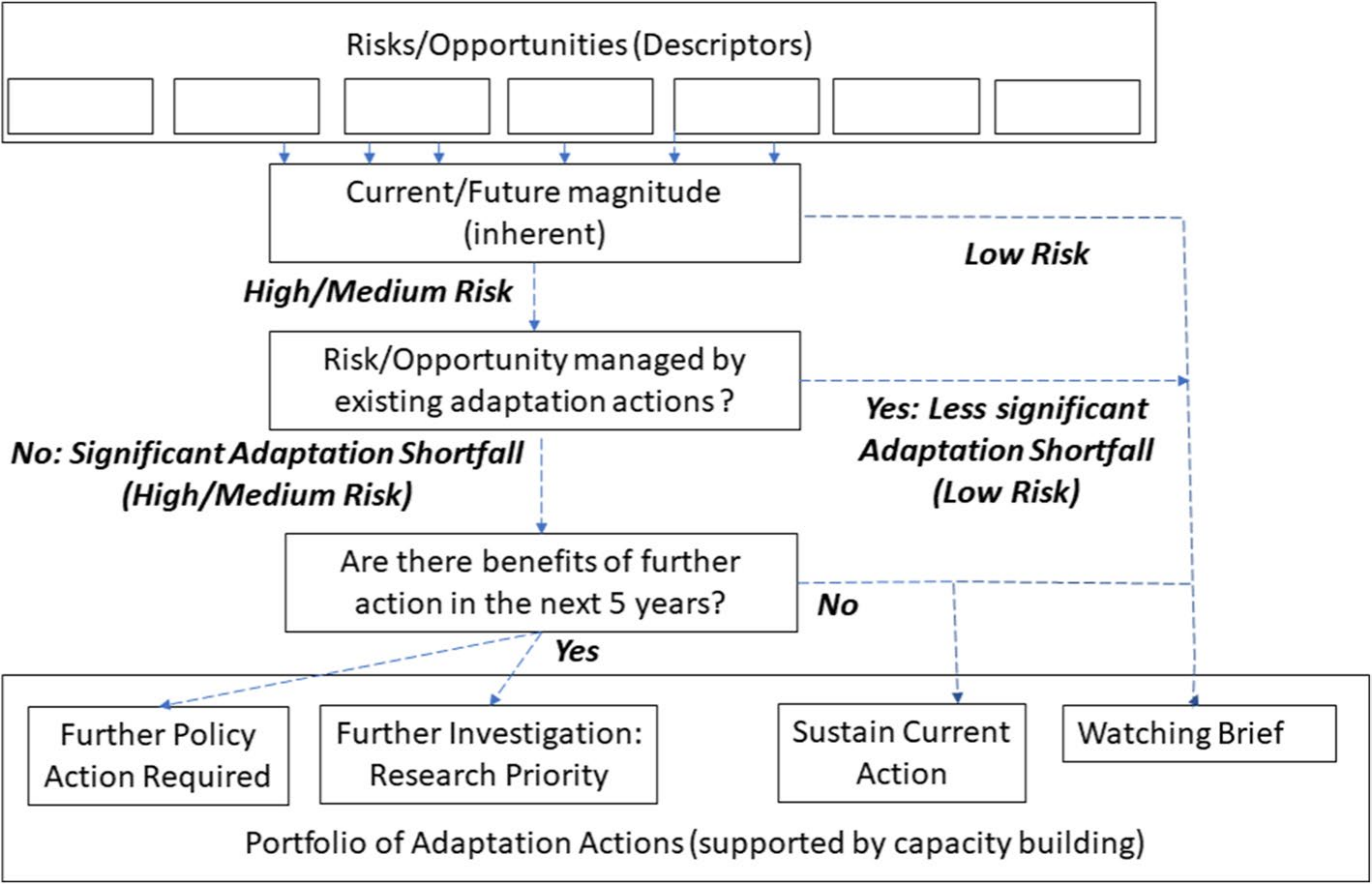
CCRA3 generic methodology flowchart (after Watkiss and Betts 2021)

As summarised above, UK CCRA generic methodologies have evolved with successive assessments, CCRA3 further refining the CCRA2 method including explicit adaptation assessment. Therefore, CCRA3 characterised risk magnitudes for present and future periods, both for inherent risk (before adaptation) and after assessing current adaptation actions (by government and other actors) in managing risk (Fig. 2). Risk magnitudes were assessed according to three categories (‘low’, ‘medium’, ‘high’) cross-referenced to different economic, social, and environmental criteria (Watkiss and Betts 2021). Adaptation was assessed against an assumed goal to manage risks to ‘low’ magnitude; any risk above this level defined a notional ‘adaptation shortfall’ providing a rationale for recommending further action (similar to ‘adaptation gap’ reporting: e.g. UNEP 2021). Qualitative confidence levels were primarily used to assess risk magnitudes based on evidence quality and consensus, similar to the IPCC schema but not invoking quantified likelihood probabilities due to consistency challenges (Mach et al. 2017).

UK CCRAs have adopted the ‘reasonable worst case’ (RWC) scenario concept to define appropriate levels of government risk preparedness, consistent with the National Risk Register (Parliamentary Office of Science and Technology 2019). For CCRA3, RWC risk magnitudes were particularly associated with a + 4 °C reference scenario (4 °C global temperature rise by 2100 compared to pre-industrial, broadly equivalent to IPCC RCP6.0 scenario) and compared to an alternative + 2 °C reference scenario that would signify major progress on climate change mitigation (broadly equivalent to IPCC RCP2.6) (Watkiss and Betts 2021).

Applying the CCRA3 method to agreed risk descriptors (Sect. 4.2) provided a common assessment format, although in practice its application varied due to diversity of risks, subjective interpretation, and evidence availability (Sect. 4.4). The methodology therefore evolved and became a focus for collective learning (both science and policy) by providing a standard template against which anomalies could be identified, although method refinements also had challenges because they had implications across the full suite of risks. Stakeholder workshops (Sect. 4.1) provided an especially suitable forum to explore method issues referenced against salient policy issues and sample evidence. Criteria to assess risk magnitudes (including opportunities) were a particularly important issue for the natural environment, with considerable debate on cross-referencing of criteria and assumed ‘acceptable’ levels of risk (i.e. ‘low’ risk magnitude).

An important CCRA3 development was moving towards a common progress-tracking framework to guide evidence interpretation on adaptation effectiveness (Watkiss and Betts 2021), as referenced against the SMART (Specific, Measurable, Achievable, Relevant, Time-Bound) protocol used for good practice in policy intervention (National Audit Office 2019). This protocol implies an implementation plan and monitoring strategy to measure policy action effectiveness (e.g. indicator-based), including robustness against multiple futures (2 °C and 4 °C reference scenarios).

For the natural environment, the generic CCRA3 methodology raised significant issues. As already highlighted, natural system complexity generally invalidates utility of simple cause-effect risk metrics. Similarly, referencing risk magnitudes against simple indicators, such as species numbers or habitat area lost, only provides crude measures of ecosystem resilience (cf. Walker 2020), notwithstanding underlying ethical issues of ‘acceptable’ biodiversity loss. A more valid proposition from a sustainability perspective is to reference risk against key factors crucial for ecosystem functioning (e.g. genetic diversity, species abundance, trophic relations, habitat patch size, and connectivity), assuming data are available. Hence, in practice, the CCRA3 narrative format was expanded to aggregate and appraise evidence from multiple contexts, including sample risk metrics, to reach an overall conclusion regarding risk magnitude with emphasis on biodiversity and healthy ecosystem function. In addition, for provisioning ecosystem services (in agriculture, forestry, and fisheries), key indicators of yield and productivity were used to reference changes in risk relative to climate and non-climate variables.

Assessing risks against effectiveness of existing adaptation actions also had difficulties, mainly due to evidential limitations (Sect. 4.4). The CCRA3 methodology implied clear distinctions between natural processes and human actions to distinguish effects of human adaptation decisions, but in practice such distinctions are often not clear in ecosystems with long histories of human interventions (Bouleau and Pont 2015). Adaptation strategies can also overlap with generic nature recovery responses. As compromise, chapter narrative structure was refined during initial inherent climate risk assessment to assess status of natural adaptation processes, distinguishing, where possible, between intrinsic capability and available opportunity (Berry et al. 2013); this established a reference path against which additional managed adaptation actions could be assessed (present and future). Risk assessment was used to highlight situations where continued degradation of biodiversity and ecosystems could lead to irreversible change, including any threshold analysis (e.g. Jones et al. 2020). Reference was also made to socioeconomic scenarios to guide interpretation of non-climate risk factors, but again with notable evidence limitations (Sect. 4.4).

Another important distinction for the natural environment against the generic methodology occurred when inferring future risk relative to a present-day baseline. For sustainability risk, this can only provide a partial assessment, as the current position typically already includes some environmental degradation, which is further exacerbated by time lags (notably as ‘extinction debt’: Kuussaari et al. 2009). Therefore, robust risk management was also conceptualised and evaluated through adaptation actions meeting sustainability objectives, consistent with current policy aspirations, to avoid ‘shifting baselines’ syndrome occurring in successive CCRAs together with implicit acceptance of ongoing incremental environmental degradation (Soga and Gaston 2018). However, further methodological development is required here to enhance rigour and consistency: for example, defining and measuring dynamic ecosystem ‘reference conditions’ associated with sustainability status remains actively debated (e.g. Bouleau and Pont 2015; Wyborn et al. 2016).

### Evidence

For credibility purposes, UK CCRAs have prioritised evidence from accessible, peer-reviewed sources (Warren et al. 2018). CCRA3 evidence was derived through multiple routes, including specialist knowledge of scientific contributors, open calls to scientific and stakeholder communities, stakeholder feedback and draft report reviews.

The natural environment has a notably large evidence base, especially for biodiversity and agriculture; hence, comprehensive evidence appraisal has logistical implications. Consequently, in CCRA3, it was impractical to formally assess each source, but instead the narrative structure aimed to provide a systematic review of all relevant evidence in terms of distinctive findings for each risk descriptor. Clearly-balanced evidence statements were particularly important for contentious issues or where new evidence suggested re-interpretation of risk magnitudes. CCRA3 evidence statements could therefore be subject to further challenge through the review process, with the published record providing a transparent log of review comments (UK Climate Risk 2021).

Evidence varied considerably between natural environment risks, with difficulties distinguishing cause-effect relations due to multiple interactions (climate and non-climate), influence of shorter-term ‘natural’ variability, and possible lagged responses or threshold effects. Confidence levels generally reduced for future periods. Consistent with other studies (Jurgilevich et al. 2017; Feng and Chao 2020; Garschagen et al. 2021), more evidence was typically available on hazard characteristics rather than spatiotemporal dynamics of exposure or vulnerability.

Evidence limitations and lower confidence levels were especially apparent for assessing adaptation actions (Table 1). Organisational reporting on climate risks and adaptation typically remains in early stages (Street and Jude 2019). Monitoring and evaluation studies, where available, were usually derived from specific spatiotemporal contexts, not necessarily representative of the national position. Policy strategies highlighted the importance of ‘climate resilience’ for adaptation but very few defined what this meant, or how it was delivered, monitored, and evaluated. Although national stakeholder surveys were available for some sectors (e.g. agriculture; forestry), and were useful in identifying barriers to adaptation, systematic evaluations of adaptation actions were usually not available. Available evidence indicated current adaptation actions remain limited regarding risk management, as shown by continuing impacts. Nevertheless, a wider knowledge base indicates reducing existing ecosystem co-stressors (e.g. pollution and toxins) and actions to particularly enhance diversity and connectivity have strong support as generic resilience strategies for nature recovery (Timpane-Padgham et al. 2017; Malhi et al. 2020).

In general, evidence constraints were greater for interpreting changing extreme events and spell lengths/sequences (dry, wet, hot, cold, etc.), both due to climate model constraints and inconsistencies between empirical and model evidence (cf. Schewe et al. 2021). Poorly referenced and inconsistent use of climate data and model projections (e.g. referencing of IPCC RCPs) were also identified by CCRA3 as hindering consistent assessment, similar to other studies (Morueta-Holme et al. 2018). Available evidence was strongly biassed towards use of IPCC RCP8.5 compared to other RCPs. Although RCP8.5 provides a valid upper-end scenario (Schwalm et al. 2021), it provides only a single scenario and at generally higher climate risk exposures than CCRA3 reference scenarios of 2 °C (especially) and 4 °C. For some evidence sources, climate risks had been estimated for 2 °C and 4 °C using analysis from RCP8.5 over a shorter time period than 2100. However, this required cautious interpretation (particularly for the 2 °C pathway), especially for extreme and threshold events that can be influenced by duration-intensity of climate forcing (Bärring and Strandberg 2018), or where risks also include GHG concentration composition (e.g. ocean acidification: Gattuso et al. 2015). Limited evidence was also available for interaction of climate with socioeconomic factors, notably changing use of land and sea, with only a few sources assessing combined climate and socioeconomic scenarios, or cross-sectoral interactions (Harrison et al. 2016).

### Scientific contributors

For the natural environment, broad diversity of risks, challenges of applying a generic methodology, and plethora of evidence sources, resulted in an intensive assessment process and the longest CCRA3 report chapter (Berry and Brown 2021). Interpretation of adaptation evidence involved a wider network of collaborators to access findings from the ‘grey’ literature because of limited academic sources. A large number of comments from peer review and stakeholder feedback were also addressed by scientific contributors (UK Climate Risk 2021). The intensity of the process considerably exceeded original estimates, and some contributors were unable to continue involvement (also exacerbated by the COVID-19 pandemic). A general conclusion was a need for an increased cadre of both specialists and generalists contributing risk-based expertise but with stronger focus on interpreting adaptation policy decision-making contexts, or otherwise to adopt a more simplified methodology.

## Key issues

Three key issues highlight evolving challenges for CCRA knowledge systems across the science-policy interface.

### Opportunities

UK CCRA3 inclusion of a more explicit assessment of opportunities together with negative risks and their net balance matches other recent national CCRAs (e.g. Federal Office for the Environment 2017). Previously, concerns have been expressed, especially by NGOs, that inclusion of adaptation opportunities would distract from the ‘main message’ on risk management. However, balanced assessment of opportunities and risks is entirely consistent with a strong focus on adaptation (New et al. 2022), supporting portfolio-based decisions that maximise positives whilst also minimising negative outcomes, both averting additional pressures on systems.

CCRA3 opportunities addressed stakeholder declared interests and perception that focusing only on negative outcomes caused disengagement from adaptation actions, sometimes manifest as a fatalistic or ‘wait and see’ culture (Mayer and Smith 2018). Opportunities therefore provided new routes for knowledge exchange, especially regarding environmental sustainability, where a positive sense of agency is fundamental to encourage behaviour change and more favourable outcomes for people and environment (Hinkel et al. 2020).

Nevertheless, including opportunities does introduce additional questions, including for methodological consistency and evidence interpretation. Moreover, trade-offs between risk and opportunities can vary according to different future pathways (climate and socioeconomic), including spatially and temporally, and whether risks/opportunities are existing or ‘emergent’. Notable examples occur through net benefits from ‘new’ species (or varieties) compared to loss of existing species for both biodiversity and for agricultural, forestry, or fisheries productivity (with implications also for greenhouse gas emissions). Hence, for CCRA3, despite original policy requests to separate out opportunities and risks, in practice this was not always feasible, particularly where broader discussion of underlying assumptions was important. An alternative approach consistent with policy requests was to distinguish risk/opportunities but not assess net effect.

Generic risk framing and terminology can be adjusted to incorporate opportunities if applied with flexibility. Hence, particularly for primary economic sectors dependent on ecosystem resources (agriculture, forestry, fisheries, etc.), opportunities materialise not only from reduced climate hazard exposure but also from climate-related enhancement of ecosystem primary productivity and therefore yields, including stability and reliability: for example, longer growing seasons or CO_2_ fertilisation effects for agriculture and forestry productivity (e.g. Arnell and Freeman 2021). Similarly, evaluating vulnerability from an opportunity perspective provided additional insights on the role of adaptive capacity, including barriers to recognition or realisation of opportunities that may perpetuate vulnerability. For example, aforementioned primary sectors often have significant legacy and path dependency issues (knowledge, skills, technology, etc.) acting against realisation of opportunities, notably for agricultural productivity (Lyle 2015).

CCRA3 found limited government awareness regarding opportunities including implicit presumption of a lesser role compared to the private sector and market forces; this overlooked intrinsic barriers, and prospective remedies through awareness-raising, knowledge exchange, and innovation support schemes (e.g. grant funding). For biodiversity policy, a default market-based rationale to realise opportunities was less apparent (although natural capital ‘markets’ are now influencing policy design: Helm 2019). Consequently, opportunities could be directly related to policy initiatives to enhance biodiversity, although presently constrained by knowledge gaps hence CCRA3 characterisation as research priorities (Table 1).

Evaluating biodiversity-related opportunities was complex in CCRA3, with ramifications of species change at ecosystem level (positive or negative) being dependent on other contextual factors. Non-native species’ colonisations may indeed provide enhanced biodiversity, but negative outcomes may also occur from invasive species disrupting existing biodiversity and ecosystem functions. A notable UK marine example is Pacific oyster (*Crassostrea gigas),* introduced commercially in southern England but now dispersing northwards. In addition to commercial catch value, Pacific oyster may potentially provide ecosystem service opportunities (e.g. water purification; habitat formation) but northerly dispersion presents a risk of loss to native oyster species (Herbert et al. 2016). In terrestrial environments, similar examples occur regarding dispersal of different tree species. For example, beech (*Fagus sylvatica)* is native to southern UK but traditionally considered a risk to native species further north and often removed from conservation areas. This position is now being re-appraised, especially due to threats to existing native tree species (e.g. Chalara disease in ash) and potential loss of beech climate space in its southern range (Yu et al. 2021). This risk/opportunity spectrum can vary spatially, and potentially also relative to climate change scenario (e.g. 4 °C versus 2 °C). Distinctions are further blurred due to cultural associations developed with ‘non-native’ species, notably a strong symbolic landscape value in some locations. Perceptions of risk or opportunity, and therefore the ‘effectiveness’ of adaptation responses, are therefore fundamentally related to which and whose values and norms are included in an assessment, as particularly evident with economic valuation of species as compared to their intrinsic value.

Dynamic interdependencies between opportunities and risks therefore remain overlooked in adaptation policy. Typically, realisation of opportunities will be contingent on effectively managing negative risks in the same (or related) systems through coordinated actions. Hence, biodiversity opportunities through new species will require good habitat condition and are dependent on actions to address current ecosystem risks (climate and non-climate). Similarly, agriculture, forestry, and fisheries opportunities require that related ecosystem risks (e.g. to soil/substrate or water quality) are successfully addressed.

### Systemic risk interactions

Risk interactions are key features of both human and natural systems: cross-sectoral, spatial or temporal, or as indirect consequences of adaptation or mitigation responses (Simpson et al. 2021). Systemic risks typically exhibit all these features, as recognised by the new ISO14091 guidance (Smith et al. 2022), manifest not just as simple additions to individual risks, but complex transboundary risk multipliers which challenge simple notions of risk ‘ownership’ (Ringsmuth et al. 2022). These features imply a greater role for coordinated government action, depending on policy position and inferred responsibilities. For the natural environment, systemic risks occur not only from chained cascading risks but through feedback cycles and threshold effects, at multiple scales from local to global, involving both direct proximal and indirect distal risk factors and adaptation/mitigation responses (Berry et al. 2014; Wassénius and Crona 2022). Although CCRA frameworks now aim to include such interactions to varying extents (Smith et al. 2022), evidence limitations continue to be a major constraint, and providing a partial sectoral assessment may significantly mis-represent systemic risks to sustainability (Harrison et al. 2016; Lawrence et al. 2020a).

For CCRA3, a systemic perspective was developed by associating individual risks through an ecosystems-based framework linking biodiversity with ecosystem services to people (Berry and Brown 2021). This framework was used to highlight synergies and trade-offs between adaptation actions (existing and additional options), together with the Net Zero agenda, notably ‘nature-based solutions’ such as restoration of woodland, wetland, peatland, coastal and marine habitats. Current UK sectoral policies potentially exacerbate systemic risks, particularly regarding land and water conflicts, and through continued reliance on hard adaptation responses that disrupt natural ecosystem responses rather than working with them (notably flood and coastal defence). Furthermore, current Net Zero plans were mainly found to omit climate risks and opportunities (including indirect effects through land-use change), meaning planned outcomes could not be considered robust future pathways. These findings highlighted a strong need to integrate adaptation policies based on provisioning ecosystem services (food; energy; water resources) within a long-term sustainability framework that provides underpinning biodiversity, ecosystem functions, regulating ecosystem services and cultural benefits. Ecosystem service provision can be defined as sustainable when demand is met without decreasing capacity for future provision of that service or causing undesirable declines in other services (Villamagna et al. 2013), requiring improved monitoring and future projections.

Much therefore remains to be done to develop a shared science-policy awareness of systemic risks. This includes improved understanding of risk factor sensitivity regarding systemic risk relationships, especially for extreme events and potential system reconfigurations, and hence also key parameters maintaining systems resilience and stability (notably ‘slow’ controlling variables: Walker et al. 2012) for different future pathways (climate and socioeconomic).

### Residual risk and risk tolerance

To better inform policy development, national CCRAs are placing increased emphasis on understanding adaptation effectiveness (Adger et al. 2018). Hence, CCRA3 used ‘adaptation shortfalls’ to prioritise urgent further actions to reduce risk to acceptable levels (Sect. 4.3). For the natural environment, concerns regarding shifting baselines meant ‘acceptable’ risk was further referenced to environmental sustainability policy objectives rather than incrementally from present positions. However, stakeholder dialogue suggested adaptation shortfalls were an unfamiliar concept, not least because objectives for successful adaptation often remain vague or ambiguous (cf. Dilling et al. 2019; New et al. 2022). By contrast, a clearer single goal for reduced GHG emissions is defined in climate mitigation policy whilst amorphous sustainability concepts are being redefined as interdependent socioecological goals (Reyers and Selig 2020).

A complementary risk communication strategy, partially explored during CCRA3, would also emphasise residual risk (i.e. unmanaged risk remaining after current adaptation actions: IPCC 2021), including any uncertainties and potential opportunities lost, and whether this would be considered ‘tolerable’. Deliberation may then lead to increased recognition of the need for further risk management actions. For example, some organisations, if not policymakers, are now being explicit regarding expectations that residual risks will increase, and for society to learn to ‘live with’ them (e.g. Environment Agency 2021). In some cases, without appropriate planning, residual risks may actually be greater than inherent risks due to the effects of maladaptation (Adger et al. 2018). This can occur with generic responses that are poorly matched to local contexts, as with flood defence schemes that shift risk downstream or downcoast; or tree-planting in the wrong location that disrupts livelihoods, damages biodiversity, and actually increases carbon emissions (e.g. on organic soils). A broader communication strategy would therefore extend beyond ‘external’ concepts of risk, as used in conventional scientific assessment, to also include ‘internal’ aspects, highlighting issues of risk perception, societal values, and risk tolerance, which are crucial in understanding options and capacity for adaptive risk governance (Dessai et al. 2004).

Increased focus on residual risk potentially raises uncomfortable issues for policy. Original guidance from UK policymakers for CCRA3 stated that scientific assessment should not make assumptions regarding societal risk tolerance, which was an issue for policy (although the UK Climate Act 2008 does not make this distinction). However, as already highlighted, many polices remain rather vague regarding target adaptation outcomes; hence, assumptions regarding residual risk and risk tolerance remain undeclared (Brown et al. 2018). CCRA3 did, however, note some implied differences between different administrations (UK and devolved), as also revealed by approaches to the COVID-19 pandemic (Ringsmuth et al. 2022).

In addition to open questions regarding assumed residual risk tolerance (including differential impacts and costs/benefits), multiple interpretations of adaptation effectiveness were implied from current policies. The normative adaptation goal of the CCRA3 generic methodology referenced standard cost–benefit policy intervention criteria (HM Treasury 2020) to prioritise reducing risks to ‘low’ magnitude (Watkiss and Betts 2021). For the natural environment, normative framing against environmental sustainability goals (stated UK policy) meant effectiveness was further interpreted through ecosystem-based adaptation to investigate synergies with natural processes. Policies also often framed adaptation as building ‘resilience’ but with insufficient details to allow evaluation. These multifaceted issues of assumed risk tolerance and adaptation effectiveness ultimately refer back to how science engages with policy development (Brown et al. 2018; Singh et al. 2021), and whether a national CCRA also aims to engage a wider audience beyond the direct policy domain to further stimulate the policy process.

To help resolve the challenges identified here, our recommendation for UK CCRA4 would indeed be to broaden its audience, and in particular to engage practitioners in addition to policymakers through forums, workshops, and surveys that aim to better understand attitudes to risk (inherent and residual) and adaptation responses. This new knowledge could in turn also inform an improved regional disaggregation of risks and cross-sectoral systemic risks.

## CCRA knowledge systems and sustainability goals

**Fig. 3:**
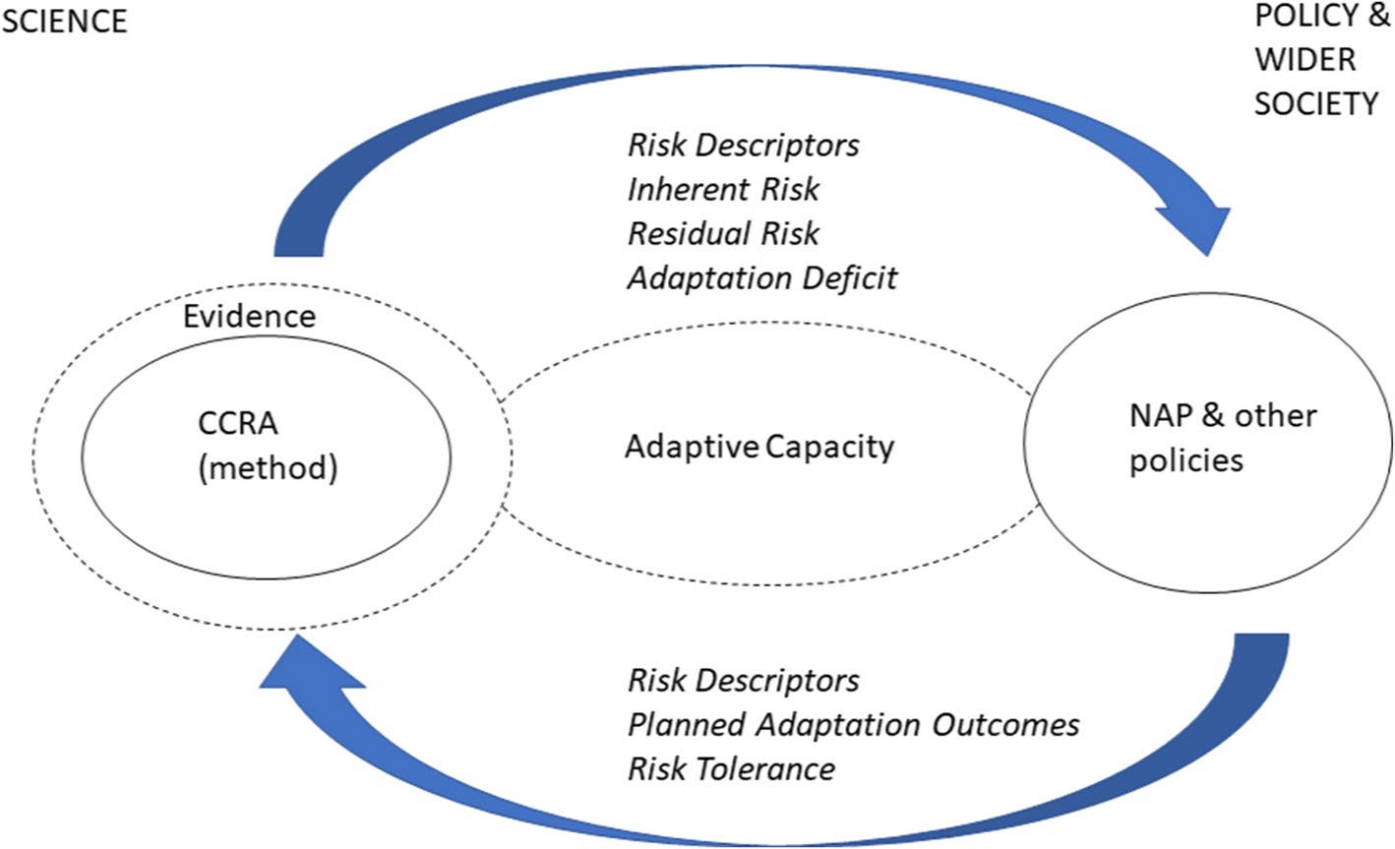
Relationships between national CCRAs and adaptation policy as dynamic iterative processes

Although adaptation is typically a complex process, interacting with evolving societal norms and values, knowledge (as distinct from information) is generally recognised as a key enabler of adaptive capacity alongside other factors (resources, risk perception, governance regime, communication, etc.; Gorddard et al. 2016; Williams et al. 2015). A knowledge system perspective emphasises open dialogue, integration, collective problem framing, plurality including diversity of values, systems thinking, and accelerated learning, recognising these will likely also be disruptive (Cornell et al. 2013; Fazey et al. 2020; Oliver et al. 2021). Regarding national CCRAs and specifically the UK, some progress on these criteria has been identified above, but rather more is required. Overall, a shift in emphasis is required from end products towards a continuing reflexive process of ‘learning by doing’ with a goal of building collective science-policy capacity for risk governance (Fig. 3), also recognising that the assessment process may itself change adaptation decision contexts (Haasnoot et al. 2020). Key findings should therefore include not only statements on risk magnitudes and adaptation effectiveness but also fresh insights and open questions on the adaptation process itself, including implications for methods, risk communication and risk ‘ownership’ across science-policy interfaces. Although adaptive capacity was implicitly included in the UK CCRA3 methodology, other national CCRAs have aimed for more explicit characterisation (e.g. Song and Lee 2021), and scope remains for further methodological development applying existing frameworks (Siders 2019).

Further progress also requires enhanced policy transparency regarding target outcomes and associated assumptions on residual risk tolerance, including deliberation with wider society as residual risks often imply a broader transformative agenda. It also requires continuing science-policy dialogue on risk framing and descriptors, reconciling scientific validity with evolving decision contexts. Target audience and participation may thus change through successive CCRA cycles, especially to decipher complex relationships of risks and adaptive responses that characterise systemic risks (Simpson et al. 2021), and to improve communication through stronger focus on agency and efficacy (McLoughlin 2021). For example, issues regarding modification of residual risk through managed coastal retreat remain confounded by barriers other than knowledge limitations (Lawrence et al. 2020b), particularly manifest in the UK at local rather than national policy level (Brown 2022).

Similar to adaptation, knowledge development for sustainability objectives is typically diverse and messy (Arnott and Lemos 2021). Challenges of integrating environmental sustainability into CCRAs match this characterisation. These challenges suggest national CCRA knowledge systems require both a pluralistic approach, to interpret risk according to differential values (e.g. Ministry for the Environment 2019), and a systems diagnostic approach to help develop and evaluate coherent, robust, adaptation policy strategies that manage risks/opportunities according to key factors (existing and emerging) (Wassénius and Crona 2022). Knowledge systems also require further development to integrate both natural and human adaptive capacity in socioecological systems including distinctions between intrinsic adaptive capability and adaptation opportunity, and hence synergies with different response strategies (Berry et al. 2013; Preiser et al. 2018). Similarly, notions of tolerable residual risk need further investigation regarding assumed stability of ecosystems and their services, including in relation to irreducible climate change uncertainties.

In scientific terms, risk magnitude and adaptation progress can be interpreted against sustainability goals at national scale through key systems properties, for example to meet fundamental human needs within ‘safe’ environmental limits defined by planetary boundaries, including candidate metrics such as human appropriation of net primary productivity (e.g. Fanning et al. 2021). Following this rationale, CCRAs would assess adaptation plans against monitored delivery of robust sustainable pathways that avoid residual risks and system instabilities particularly associated with thresholds and tipping points (Bauch et al. 2016; Haasnoot et al. 2018). In policy terms, this would be consistent with ongoing application of the ‘Precautionary Principle’ to prioritise actions that anticipate and avoid future environmental harm despite inherent scientific uncertainty (as defined in international agreements: UNESCO 2005). In practical terms, this requires a greater emphasis in CCRA knowledge systems on concepts of ‘what works, where and when’ regarding both adaptation and sustainability objectives (Runhaar et al. 2018), including synergies with climate mitigation actions.

A knowledge systems agenda also defines priorities in terms of connected policy and research actions. In UK CCRAs, despite CCRA3 developments, findings have tended to be seen as directed at distinct policy and science communities, rather than shared portfolio of actions. This has meant strategic policy and research interactions are overlooked, notably for systematic monitoring and evaluation of existing adaptation actions and cross-sectoral systemic risks. In sustainability terms, linkages may be facilitated by an increased emphasis on research to enable adaptive change rather than just research about change, highlighting additional beneficial actions to reach defined objectives (Fazey et al. 2020; McLoughlin 2021).

CCRA knowledge systems could also be enhanced through increased emphasis on ‘living-evidence’, regularly incorporating new research findings into evidence syntheses (Elliot et al. 2021), as exemplified by current ‘impact report cards’ (e.g. MCCIP 2020), but with stronger focus on implications for adaptive management. This requires appropriate resources and planning, together with an institutional culture that better recognises topicality and intellectual importance of evidence syntheses (Donnelly et al. 2018). Reappraisal of evidence, and particularly new types of evidence, may enhance risk confidence assessments and indicators of adaptation progress (Pearce-Higgins et al. 2022).

Current CCRAs have identified crucial knowledge gaps. As found by CCRA3, knowledge on exposure and vulnerability dynamics of risk is typically less developed than on hazard, especially for extreme or compound events, threshold effects, and systemic risks (Jurgilevich et al. 2017; Zscheischler et al. 2018; Garschagen et al. 2021). For natural environment risks, improved referencing against climate scenarios is required (Morueta-Holme et al. 2018), especially regarding availability and interpretation of bioclimate rather than primary climate data (Brown 2018). Increased emphasis on sustainability risk also requires improved cross-scale referencing of robustness of adaptation pathways against non-climate risk factors, including through socioeconomic scenario frameworks and the dynamics of socioecological vulnerability (Frame et al. 2018). For example, a 2 °C scenario pathway assumes major global climate mitigation activities, including through nature-based solutions, with consequent implications regarding co-evolution and effectiveness of adaptation responses.

## Conclusion

National CCRAs face distinctive challenges that differ from a conventional science-based risk or policy assessment. Framing of risks against adaptation contexts is complicated by their multiple facets and interpretations. CCRAs need to reconcile different influences across the science-policy interface, especially regarding risk descriptors, methodology, and evidence sources. Challenges are particularly pronounced for the natural environment, because of intrinsic complexity and diversity of contexts, but environmental sustainability goals can provide a unifying theme.

Approaches to integration and enhanced risk communication were explored by reference to the UK CCRA, with current findings showing that adaptation actions incur a major shortfall compared to requirements for establishing a robust pathway to environmental sustainability. A knowledge systems perspective was used to diagnose and make progressive recommendations in the context of adaptive risk governance. This highlights national CCRAs as evolving, transdisciplinary processes that are open, reflexive, and ultimately adaptive to changing decision contexts, influenced by both knowledge advances and evolving societal values, particularly regarding interpretation of risk and adaptation in the overall context of sustainability goals. Key issues have been identified regarding inclusion of opportunities, systemic risks, residual risks, adaptation effectiveness, and assumed risk tolerance. Assessing robust pathways to sustainability requires improved explication of both risk and uncertainty of outcomes in relation to societal expectations. Addressing this knowledge agenda requires innovative risk framing and methodological developments to assess sustainability risk, including dynamic reference conditions and consistency with climate mitigation (Net Zero) goals. It also requires transparent policy objectives that can be iteratively investigated against multiple climate and socioeconomic futures. For environmental sustainability, this would include further development and application of the Precautionary Principle in the face of climate change.

## Data Availability

Data sharing not applicable to this article as no datasets were generated or analysed during the current study.
